# The Human Shield in Time but Not in Space: Scale-Dependent Responses of Small Indian Civet–Prey Interactions to Anthropogenic Disturbance

**DOI:** 10.3390/ani15213121

**Published:** 2025-10-28

**Authors:** Chengpeng Ji, Xiaochun Huang, Yufang Lin, Yanan Cheng, Tongchao Le, Fanglin Tan

**Affiliations:** 1 Fujian Academy of Forestry, Fuzhou 350012, China; toncher@163.com (T.L.); fanglintan@163.com (F.T.); 2 Fujian Forestry Prospect and Design Institute, Fuzhou 350001, China; hxc13559326373@163.com (X.H.); yaming711@163.com (Y.C.); 3 Fujian Liangye Mountain National Nature Reserve Management Bureau, Wuping 364300, China; linyufanglyes@163.com

**Keywords:** human activity, small Indian civet, predator–prey, spatiotemporal overlap, camera trapping

## Abstract

**Simple Summary:**

Human activities are increasingly altering natural ecosystems, but their effects on predator–prey interactions are not fully understood. We used camera traps to study how human activity (human presence, roads, and settlements) as well as altitudes and seasons influence the spatiotemporal relationships between the small Indian civet and its potential prey (nocturnal rats, diurnal Pallas’s squirrels, and Chinese bamboo partridges) in southeastern China. Our results show that human disturbance caused both predators and prey to shift their daily activity patterns. Temporally, high human activity provided diurnal prey with a refuge from small Indian civet predation, supporting the “human shield” hypothesis. However, spatially and spatiotemporally, higher human disturbance increased overlap between small Indian civets and prey, potentially raising encounter rates and predation risk. These findings demonstrate that human impacts on wildlife interactions are scale-dependent, underscoring the necessity of multi-dimensional approaches in conservation planning.

**Abstract:**

Despite growing evidence of the widespread impacts of human activities on carnivores and their prey, it remains unclear how different types and intensities of human disturbance reshape predator–prey interactions. In this study, we conducted a systematic camera-trapping survey on a threatened carnivore, the small Indian civet (*Viverricula indica*). This species forages on prey with contrasting diel patterns, including nocturnal rats and diurnal species such as Pallas’s squirrel (*Callosciurus erythraeus*) and Chinese bamboo partridge (*Bambusicola thoracica*) in the southern Wuyi Mountains of southeastern China. Based on data from an extensive sampling effort (60,901 trap days at 180 camera stations), we used kernel density estimation and Pianka’s index to examine whether and how different types and intensities of human activity (human presence, roads, and settlements), as well as diverse altitudes and different seasons, affect the spatiotemporal interactions between small Indian civets and their potential prey. We found that all studied species adjusted their activity patterns, either advancing or delaying their peaks, to achieve temporal segregation under varying types and intensities of human disturbance and different altitudes and seasons. At the temporal scale, interactions between small Indian civets and their potential prey supported the human shield hypothesis, suggesting that increased human disturbance provides diurnal prey with refuge from predation pressure. Conversely, at both spatial and spatiotemporal scales, higher levels of human disturbance increased the overlap between small Indian civets and their prey species. These findings highlight that human impacts on wildlife interactions are scale-dependent: temporal refuge for prey does not necessarily reduce spatial or spatiotemporal overlap, which may still increase encounter rates and predation risk. Because our sampling relied on ground-level cameras, our inferences are limited to terrestrial interactions; arboreal interactions remain unquantified and require combined ground–canopy sampling in future work. Effective conservation management thus requires considering these scale-dependent effects of human activities on wildlife interactions.

## 1. Introduction

Due to continuous pressures from human activities, competition between humans and wildlife for spatial and temporal resources is intensifying. This competition results in various direct and indirect impacts on wildlife distribution, activity patterns, reproduction, and survival [1], seriously threatening global biodiversity conservation and ecosystem functions [2,3]. However, animal responses to human activities are rarely uniform [4]. These behavioral shifts may further change their temporal and spatial overlap, which in turn may either strengthen or weaken the strength of interspecific interactions [5,6,7]. As key components of the food chain, carnivores directly or indirectly shape prey population structures through predation and intimidation [8]. However, due to their large spatial requirements, they are particularly vulnerable to human activities, which can potentially alter predator–prey relationships and, in turn, affect ecosystem stability [9,10]. Thus, understanding the effects of human activity on predator–prey interactions is essential for testing hypotheses regarding the diverse ways that increasing human activity may affect wildlife persistence and coexistence [8,11].

The premise for the predator–prey interaction to occur is that the predator and prey must co-occur in space and time [12]. Predators must synchronize their activity patterns with those of their primary prey, likely reducing energy expenditure during hunting [13]. Conversely, prey species often employ antipredator strategies to evade detection or capture, such as adjusting their spatial distribution or timing of activities to minimize overlap with predators, especially in areas where predation risk is relatively predictable [14]. However, human activities can alter the activity patterns and habitat use of both predators and prey, potentially increasing or decreasing their temporal and/or spatial overlap and thus affecting encounter probabilities [2,15].

Previous studies suggest that when predators and prey exhibit similar behavioral responses—either both avoiding or both being attracted to human activities—their encounter likelihood increases, potentially favoring predators [16], and the loss of spatiotemporal refuges, which previously contributed to stable predator–prey coexistence, may also increase predation rates [17]. Conversely, when predators and prey exhibit opposing responses to human activities—for instance, predators respond to human activities by altering their movement, activity patterns, or resource use [3,18], while prey actively seek out human-dominated areas for resources or protection [19], known as the human shield hypothesis [20]—their likelihood of encounters decreases, potentially giving prey the advantage [21]. Thus, the effects of human activities on predator–prey interactions are complex and context-dependent. Understanding this variability is crucial for predicting shifts in predator–prey dynamics and their potential cascading effects on trophic structures [15].

Different types of human activity may have various effects on wildlife spatiotemporal interaction [11,22], which are often overlooked in previous studies. For instance, predators may avoid human infrastructure (e.g., roads and settlements), while prey may increase their use of these areas to access resources or reduce predation risk, as predicted by the human shield hypothesis [19]. However, short-term, relatively regular disturbances, such as human presence, may primarily prompt shifts in the temporal activity patterns of predators and prey [22]. In China’s central Taihang Mountains, wild boars (*Sus scrofa*) used roads as refuges to avoid predation by leopards (*Panthera pardus*) [7]. In West Africa, lions (*P. leo*) and spotted hyenas (*Crocuta crocuta*) increased their temporal overlap with three prey species due to their prey shifting their nocturnal activity to avoid human presence [13]. In addition to disturbance type, the seasonal dynamics of human activities also exert significant influences on predator–prey interactions, as humans are more likely to hike or engage in recreational activities in forests during cooler seasons, thereby posing seasonal threats to predator–prey relationships [23,24]. Therefore, disentangling the simultaneous effects of multiple types and seasonality of human activity is essential for a comprehensive understanding of their impacts on predator–prey interactions and the subsequent ecological consequences.

The small Indian civet (*Viverricula indica*) is mainly distributed in the central, southern and southwestern parts of China [25]. It is a nocturnal and crepuscular omnivore, inhabiting a range of forested and human-modified landscapes, where it forages for small vertebrates and plant matter. However, severe habitat loss and illegal hunting over the past few decades have led to population declines of small Indian civets, posing a major challenge to the conservation of this species [26]. Research on small Indian civets in the wild has focused on their activity patterns, habitat use, and responses to human activities [27,28,29,30], interspecific competition and coexistence with sympatric carnivores [31,32,33,34,35], and the selection of potential prey resources across different seasons [36]. Previous studies indicate that small Indian civets tend to avoid areas of high human activity, though they may also use human-modified landscapes for foraging [27,28]. However, there is a lack of reports on the interaction between the small Indian civets and their potential prey under multiple types and intensities of human disturbance.

In the hilly and mountainous regions of southeastern China, with the continued expansion of human activities, large carnivores have gone extinct, making the small Indian civets the top predator in the local food web. The population of small Indian civets may be facing dual pressures from intense human activities and mesopredator release, which could potentially lead to population collapse. The mesopredator release hypothesis states that the extinction of large carnivores often leads to the population expansion of small- and medium-sized predators [37]. Thus, to investigate the effects of different types and intensities of human disturbance on the spatiotemporal relationships between small Indian civets and their potential prey, which differ in diel activity patterns—nocturnal rats and diurnal prey (i.e., Pallas’s squirrels (*Callosciurus erythraeus*) and Chinese bamboo partridges (*Bambusicola thoracica*))—we conducted a camera-trapping survey in the southern Wuyi Mountains, southeastern China. The Wuyi Mountains are also a priority area for biodiversity conservation in China; however, predator–prey relationships in this region have rarely been reported. Using an extensive dataset from 180 camera sites, we examined whether and how types and intensities of human activity (human presence, roads and settlements), diverse altitudes and different seasons affected the spatiotemporal interactions between small Indian civets and their potential prey. We developed the following contrasting hypotheses regarding how human disturbance would affect spatiotemporal overlap: H1: The Spatial Refuge Hypothesis. We hypothesized that roads and settlements would serve as spatial refuges for diurnal prey [7] but not for small Indian civets. This would lead to an overall reduction in spatiotemporal overlap between small Indian civets and diurnal prey [21]. As a compensatory foraging strategy, we expected an increase in spatiotemporal overlap between small Indian civets and nocturnal rats. H2: The Activity Compression Hypothesis. We hypothesized that high levels of human presence would cause both small Indian civets and diurnal prey to shift their activity into crepuscular or nocturnal hours [15]. This behavioral convergence would lead to an increase in temporal, and consequently spatiotemporal, overlap between them [38].

## 2. Materials and Methods

### 2.1. Study Area

This study was conducted in the Fujian Liangye Mountain National Nature Reserve (116°07′–116°19′ E, 25°04′–25°20′ N), Wuping County, Longyan City, Fujian Province, China. It covers an area of 143.65 km^2^, with an elevation from 273 m to 1538.4 m. Located at the southernmost end of the Wuyi Mountain and adjacent to the Nanling Mountains in Guangdong Province, the reserve lies in the transitional zone between the mid-subtropical and south-subtropical regions. This area has a typical subtropical monsoon climate, with an average annual temperature of 17.0–19.6 °C, an extreme minimum temperature of −6.3 °C, and an extreme maximum temperature of 38 °C. The average annual precipitation is 1706.5 mm, the average relative humidity is 78%, and the frost-free period lasts approximately 278 days per year [39]. The vegetation exhibits a distinct vertical gradient, transitioning from evergreen broad-leaved forests at lower elevations to evergreen coniferous and broad-leaved mixed forests, and finally to montane mossy dwarf forests at higher elevations [39]. The reserve preserves a representative mid-subtropical forest ecosystem, with a forest coverage rate of 88.4%. Its flora and fauna display typical characteristics of transitional zones, and its complex geographical conditions support rich biodiversity resources [40]. The reserve is surrounded by several small rural settlements (population < 300 each), primarily located in valleys and along roads. Human activities include farming and limited tourism, with most settlements concentrated in the central and southern parts of the reserve. A network of roads was constructed to improve residents’ mobility and to support forest-fire prevention.

### 2.2. Camera-Trapping Survey

**Figure 1:**
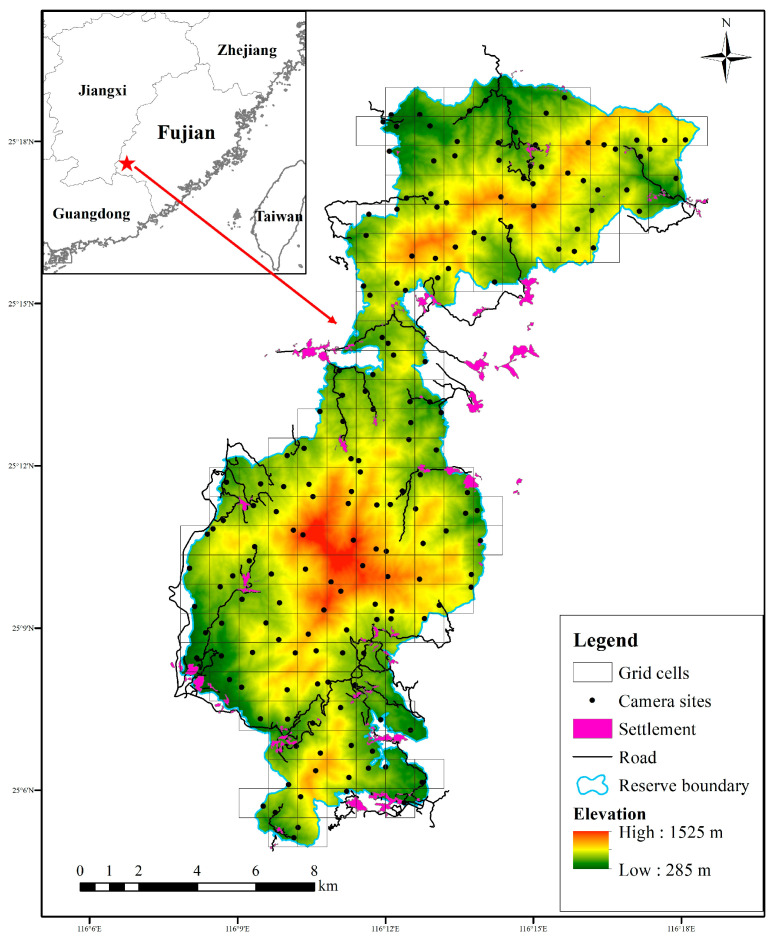
Camera-trapping sites in Fujian Liangye Mountain National Nature Reserve.

We divided the whole reserve into a grid of 1 × 1 km cells by using the fishing net tool in ArcGIS 10.2, making use of the distribution and availability of dirt roads inside grids. We positioned one unbaited camera trap (ERE-E3H-C, Shenzhen Ereagle Technology Co., Ltd., Shenzhen, China) along roads and wildlife trails within each grid from September 2022 to October 2023. In total, 181 camera traps were installed, and adjacent camera sites were at least 300 m apart in the reserve (Figure 1), and all cameras were operational simultaneously, allowing continuous data collection across the study period. The cameras were fastened to trees or bushes 0.3–0.6 m above the ground and programmed to capture three consecutive photographs followed by a 15 s video for each triggering event, with a trigger interval of 0 s between consecutive events, using medium sensitivity settings for optimal results [41]. In order to avoid false triggering, the vegetation around each camera trap was cleared wherever necessary. The camera traps were set to operate for 24 h a day throughout the year. Environmental information of each camera site was recorded, such as installation time, GPS location, elevation, and forest types. We visited each camera trap every three months to check the operating state and replace the memory cards and batteries. Camera traps in each grid cell were also replaced if broken or lost.

### 2.3. Data Analysis

Excluding a missing camera in one grid, we ultimately analyzed the data from 180 camera traps. If the camera failed during shooting in some grids, the last photograph taken was determined to be the final operational date [42]. Due to the difficulty in distinguishing the rats taken by the camera traps in most cases, they were grouped as “nocturnal rats” in subsequent analyses [43]. When one or multiple photographs and videos of the same species were taken within a 0.5 h interval, they were considered as an independent detection to avoid pseudoreplication, regardless of whether the same or different individuals were captured [44].

#### 2.3.1. Daily Activity Pattern and Temporal Overlap

The kernel density estimation method was used to describe the daily activity pattern of small Indian civets and their potential prey [45]. We pooled independent detections from all camera traps to estimate the daily activity patterns of small Indian civets and their potential prey. Firstly, the raw clock time was converted to true solar time to eliminate the effect of changes in day length during the survey period [46]. Then, the solar times were transformed into radians (2π = 24 h) before fitting a kernel density function to calculate the daily activity pattern of species with the “overlap” package in R (4.4.1) [45].

We performed pairwise comparisons of the activity patterns between small Indian civets and their potential prey by using the overlap coefficient (∆), which measures the area under the curve shared by the two functions compared and ranges from 0 (no overlap) to 1 (complete overlap). Overlap indices were calculated using ∆_4_ (independent detections were 75 or greater than 75) and ∆_1_ (independent detections lower than 75) equations according to the sample sizes obtained [45]. Moreover, we evaluated differences in pairwise comparisons of daily activity patterns between small Indian civets and their potential prey using the nonparametric Watson–Wheeler test with the “circular” package in R [47]. The level of significance was set at *p* < 0.05. Overlap coefficients and their respective 95% confidence intervals were calculated with the “overlap” package in R.

We also examined whether the temporal interaction between small Indian civets and their potential prey shifted due to human disturbances, altitudes and seasons. We described the potential effects of human disturbance using variables such as human presence and the distance from the camera site to the nearest settlement and major road. The human presence estimates were the number of human activities (all people on foot and domestic animals) detected per 100 camera-trap days on a given camera site, averaged across the study period. Data on distance to the nearest settlement and road were obtained from OpenStreetMap for the year 2023 and were calculated in ArcGIS 10.2 using Euclidean distance. To quantify how human disturbances, altitudes and seasons influence temporal overlap between small Indian civets and their potential prey, we grouped camera stations into three classes for each variable by using the first and third quartiles of the distribution for data splitting [48]. For human presence, we split the distribution into three distinct effect segments: low (<1.98 detections per 100 trap days; *n* = 71), moderate (1.98–4.39; *n* = 51), and high (>4.39; *n* = 58). Distance to the nearest settlement was classified as near (<500 m; *n* = 50), moderate (500–1000 m; *n* = 55), and far (>1000 m; *n* = 75). Distance to the nearest road was classified as near (<400 m; *n* = 77), moderate (400–1000 m; *n* = 58), and far (>1000 m; *n* = 45). Altitude was binned into low (<500 m; *n* = 47), moderate (500–800 m; *n* = 86), and high (>800 m; *n* = 47). The warm and cold seasons were defined according to the average daily temperature and day length. The warm season (May to October) had an average temperature of 34.6 °C, while the cold season (the remaining months) averaged 20.5 °C [42].

#### 2.3.2. Spatial Distribution Overlap

We calculated a spatial overlap index (Pianka’s index) [49] to investigate the spatial overlap between small Indian civets and their potential prey based on the relative abundance index (RAI) data. The RAI was the number of detections per 100 camera-trap days for each species in different camera stations [44]. Pianka’s index ranges from 0 (no overlap) to 1 (complete overlap). Pairwise species spatial overlap analysis and bootstrapped confidence intervals were calculated with the “spaa” package in R [50].

We also quantified spatiotemporal overlap using the product of temporal overlap and spatial overlap to represent the probability of the small Indian civet selecting potential prey both in space and time [51].

## 3. Results

**Figure 2:**
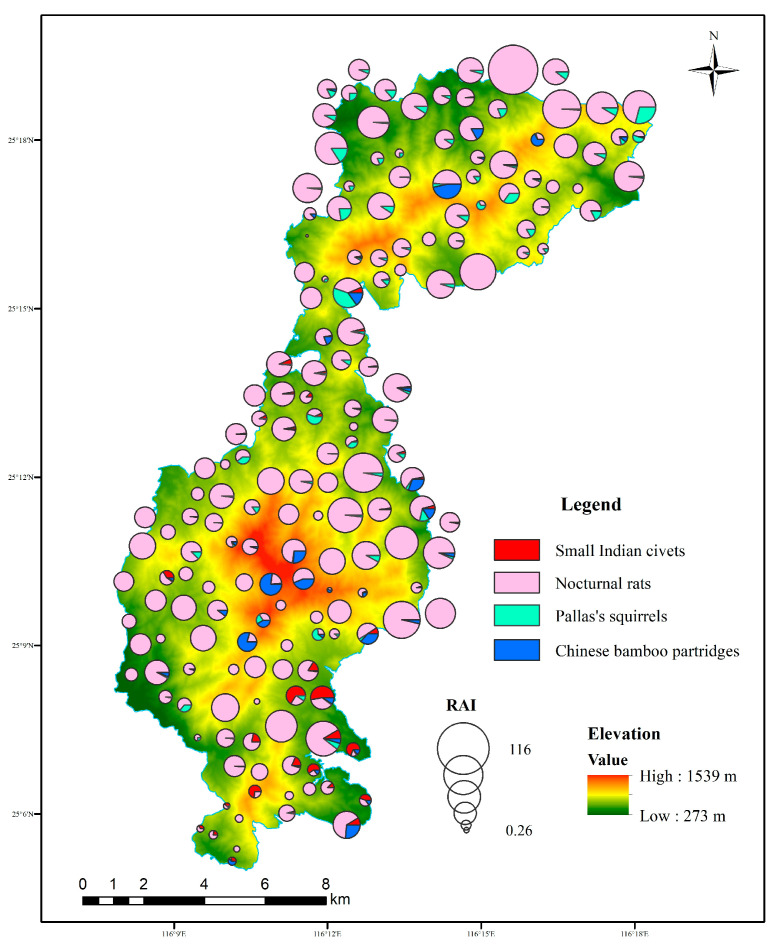
Spatial distributions of small Indian civets and their potential prey and the composition of RAI in each camera site in Liangye Mountain National Nature Reserve.

During the study periods, a total of 43,888 independent photos of mammal and bird species were taken from 60,901 trap days across 180 camera traps, including 307 detections of small Indian civets, 10,429 detections of nocturnal rats, 518 detections of Chinese bamboo partridges, and 629 detections of Pallas’s squirrels (Table 1). Nocturnal rats were widely distributed and detected at 179 sites in the study area, followed by Pallas’s squirrels and Chinese bamboo partridges. The RAI of nocturnal rats was the highest, followed by Pallas’s squirrels, but that of small Indian civets was the lowest. Small Indian civets were mainly recorded in the central and southern parts of our study area (Figure 2).

### 3.1. Daily Activity Pattern

**Figure 3:**
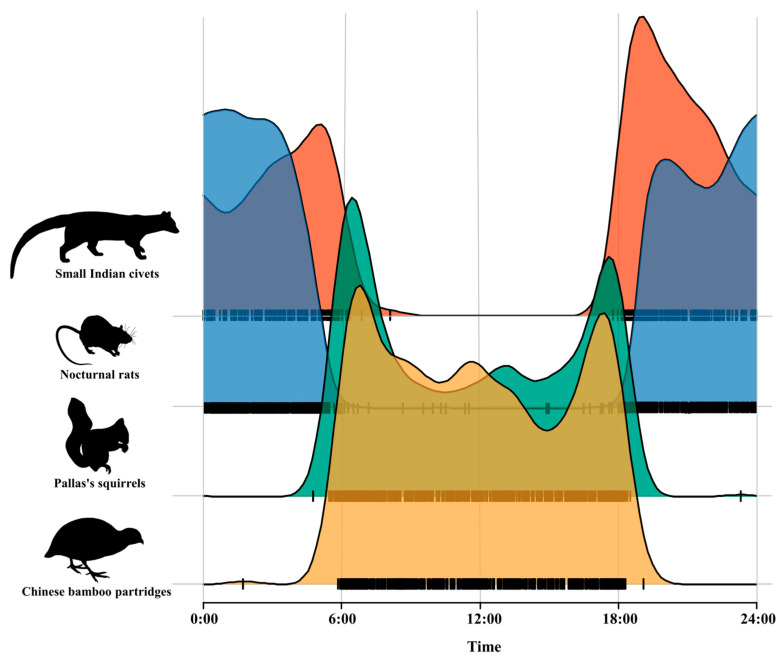
Daily activity pattern of small Indian civets and their potential prey in Fujian Liangye Mountain National Nature Reserve.

Kernel density estimation of the daily activity pattern of small Indian civets and their potential prey revealed that small Indian civets and nocturnal rats showed nocturnal activity patterns, while Pallas’s squirrels and Chinese bamboo partridges showed diurnal activity patterns. The daily activity patterns of small Indian civets and their potential prey were bimodal, exhibiting two distinct peaks. The activity peak of small Indian civets occurred around 04:00–06:00 and 18:00–20:00, with significantly reduced activity at 06:00–18:00. The activity peak of nocturnal rats appeared during 19:00–21:00 and 00:00–02:00. Pallas’s squirrels and Chinese bamboo partridges were more active in the early morning (06:00–07:00) and at dusk (17:00–18:00), but their activity peaks were slightly different (Figure 3).

Kernel density overlap indices indicated that the temporal overlap between small Indian civets and nocturnal rats was the highest (Δ = 0.72), followed by Pallas’s squirrels (Δ = 0.20), with Chinese bamboo partridges (Δ = 0.16) being the lowest pair values.

### 3.2. Temporal Overlap Under Different Human Disturbances

Daily activity patterns of the small Indian civet and its potential prey varied along gradients of human disturbances, as reflected by human presence, distance to settlement, and distance to road. Under moderate to high human encounter intensities, small Indian civets reduced their activity around sunset and increased their activity during early morning hours. In contrast, nocturnal rats exhibited no significant changes in activity across varying human presence levels. Pallas’s squirrels showed increased activity at sunrise and decreased activity at sunset under moderate human presence, whereas this pattern was reversed under high human presence. Chinese bamboo partridges peaked in activity around noon under low to moderate human presence but shifted to dawn and dusk peaks under high human presence (Figure S1).

**Figure 4:**
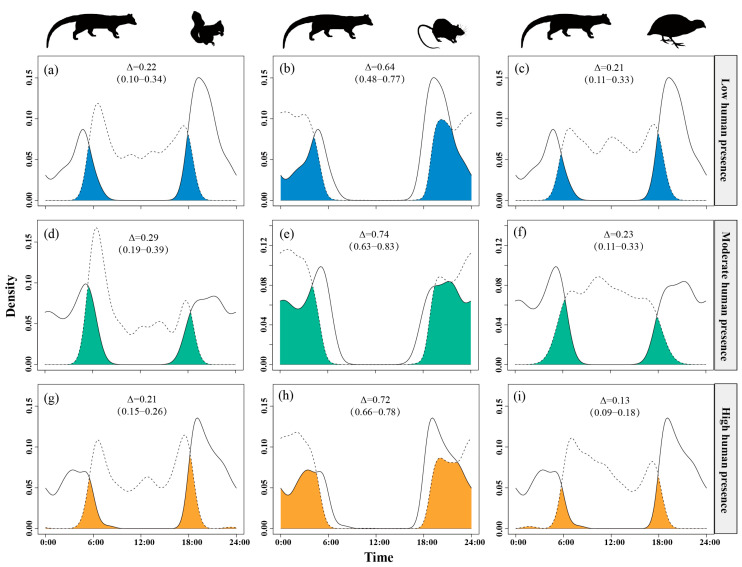
The temporal overlap between small Indian civets (solid curves) and their potential prey (dashed curves) in areas of low (**a**–**c**), moderate (**d**–**f**) and high (**g**–**i**) levels of human presence in Fujian Liangye Mountain National Nature Reserve. Shaded area denotes the overlapping time under the two density curves (Δ).

Temporal overlap between small Indian civets and their potential prey varied across gradients of human presence. The overlap with nocturnal rats remained consistently high (Δ = 0.64–0.74) across all disturbance levels. The temporal overlap with Pallas’s squirrels was moderate under moderate human presence (Δ = 0.29) but was lower at both low and high levels of human presence (Δ ≈ 0.21–0.22). Overlap with Chinese bamboo partridges was consistently low (Δ = 0.13–0.23), with further reductions observed as human presence intensity increased (Figure 4).

At a moderate distance from the settlement, small Indian civets exhibited a unimodal activity pattern, with peak activity occurring during the first half of the night. In contrast, both close and far distances were characterized by a bimodal crepuscular pattern, with activity peaks in the morning and evening that occurred earlier at greater distances. Nocturnal rats displayed no significant variation in activity across the distance gradient. For Pallas’s squirrels, increasing distance from settlements was associated with greater early-morning activity intensity and advance in activity timing, whereas daytime activity decreased at moderate distances. Chinese bamboo partridges advanced their early-morning activity peaks at moderate and far distances, with increased morning activity and reduced evening activity at moderate distances. At far distances, daytime activity decreased while evening activity increased (Figure S2).

**Figure 5:**
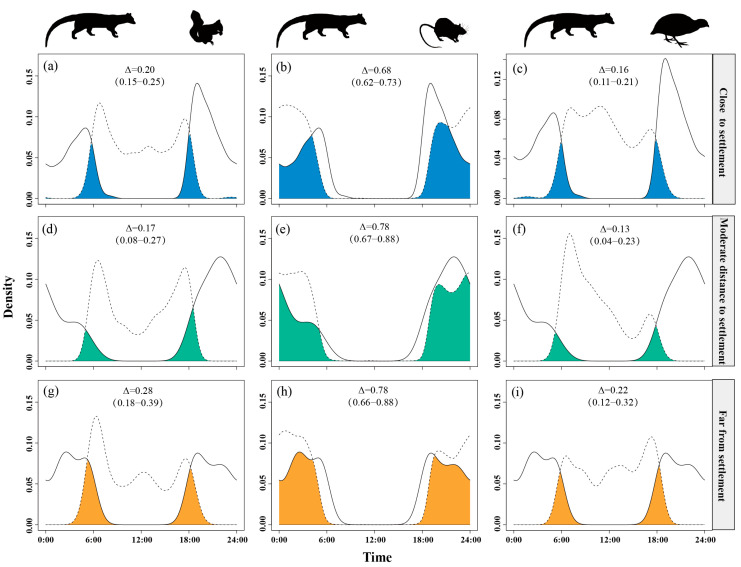
The temporal overlap between small Indian civets (solid curves) and their potential prey (dashed curves) in areas of close (**a**–**c**), moderate (**d**–**f**) and far (**g**–**i**) distance to settlement in Fujian Liangye Mountain National Nature Reserve. Shaded area denotes the overlapping time under the two density curves (Δ).

Temporal overlap between small Indian civets and nocturnal rats was lower in areas close to settlement (Δ = 0.68) but higher at moderate and far distances (Δ = 0.78). Overlap with Pallas’s squirrels remained moderate (Δ = 0.17–0.28), peaking at the farthest distances (Δ = 0.28). Overlap with Chinese bamboo partridges was consistently low (Δ = 0.13–0.22), reaching the lowest level at moderate distances (Δ = 0.13) (Figure 5).

At greater distances, small Indian civets advanced their morning activity peak and delayed their evening activity peak compared to sites closer to roads. At moderatedistances, morning peaks were delayed while evening peaks advanced. Nocturnal rats showed no significant variation in activity across the distance gradient. Pallas’s squirrels displayed a bimodal activity pattern close to and at moderate distances from roads but shifted to a trimodal pattern farther away, adding a midday activity peak and showing stronger early-morning activity farther from roads. Chinese bamboo partridges delayed morning peaks and advanced evening peaks at moderate distances, whereas at greater distances, morning activity advanced and evening activity delayed (Figure S3).

**Figure 6:**
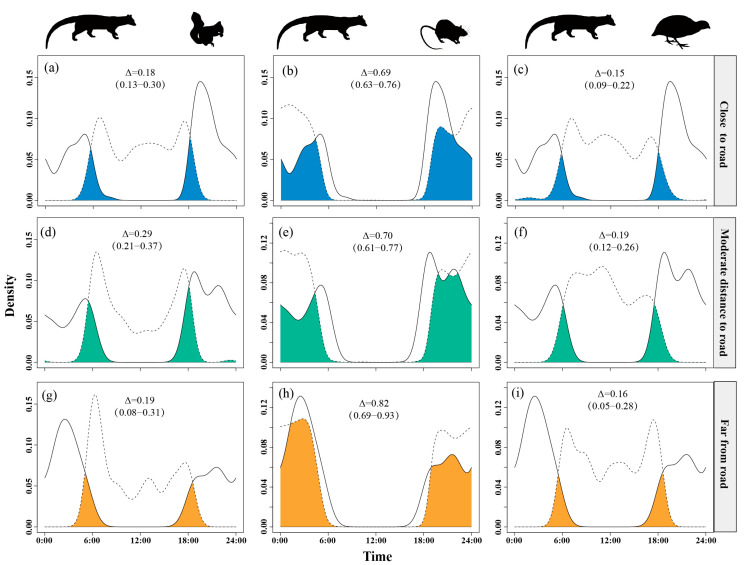
The temporal overlap between small Indian civets (solid curves) and their potential prey (dashed curves) in areas of close (**a**–**c**), moderate (**d**–**f**) and far (**g**–**i**) distance to road in Fujian Liangye Mountain National Nature Reserve. Shaded area denotes the overlapping time under the two density curves (Δ).

Temporal overlap between small Indian civets and nocturnal rats significantly increased as distance from roads increased, reaching its peak (Δ = 0.82) at the farthest sites. Overlap with Pallas’s squirrels was intermediate at moderate distances (Δ = 0.29) but lower near (Δ = 0.18) and far from roads (Δ = 0.19). Overlap with Chinese bamboo partridges remained consistently low (Δ = 0.15–0.19), with a slight increase at moderate distances (Δ = 0.19) (Figure 6).

### 3.3. Temporal Overlap Under Different Altitudes and Seasons

Daily activity patterns of the small Indian civets and their potential prey varied across altitude gradients and between seasons. At higher altitudes, small Indian civets exhibited earlier morning activity peaks and delayed evening peaks compared to lower sites. Nocturnal rats showed no significant differences in activity timing across altitudes. Pallas’s squirrels maintained relatively stable morning and evening peaks, with an additional afternoon activity peak emerging at lower altitudes. Chinese bamboo partridges displayed largely consistent morning and evening activity timing across altitudes, although a noticeable afternoon activity trough occurred at mid-altitude sites (Figure S4).

**Figure 7:**
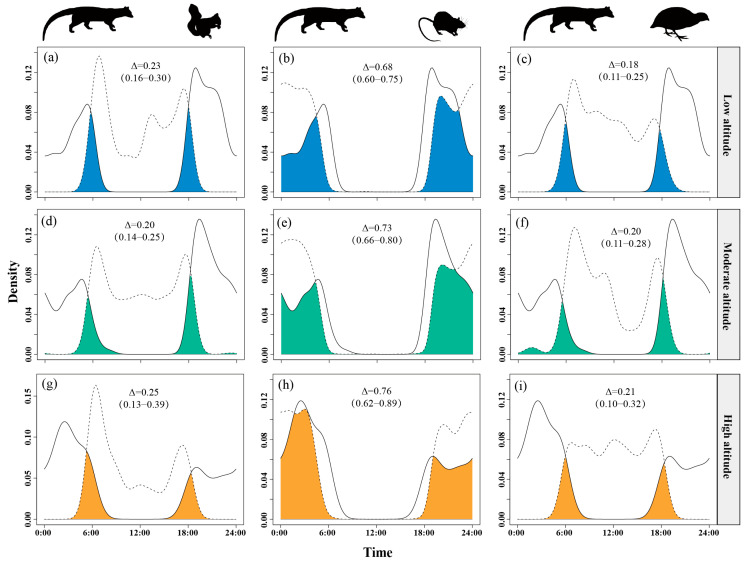
The temporal overlap between small Indian civets (solid curves) and their potential prey (dashed curves) in areas of low (**a**–**c**), moderate (**d**–**f**) and high (**g**–**i**) altitude in Fujian Liangye Mountain National Nature Reserve. Shaded area denotes the overlapping time under the two density curves (Δ).

Temporal overlap between small Indian civets and nocturnal rats increased with altitude, from Δ = 0.68 at low altitudes to Δ = 0.76 at high altitudes, peaking at the highest sites. Overlap with Pallas’s squirrels was moderate, slightly higher at high altitudes (Δ = 0.23–0.25) but lower and similar at low and mid-altitudes (Δ = 0.18–0.20). Overlap with Chinese bamboo partridges remained consistently low (Δ = 0.18–0.21) across all altitude levels (Figure 7).

During the warm season, small Indian civets delayed their morning activity peak and advanced their evening peak, accompanied by increased morning activity intensity. Nocturnal rats showed a similar delay in morning activity but reduced evening activity intensity. Pallas’s squirrels also delayed morning peaks and advanced evening peaks, whereas Chinese bamboo partridges increased midday activity and reduced evening activity during the warm season (Figure S5).

**Figure 8:**
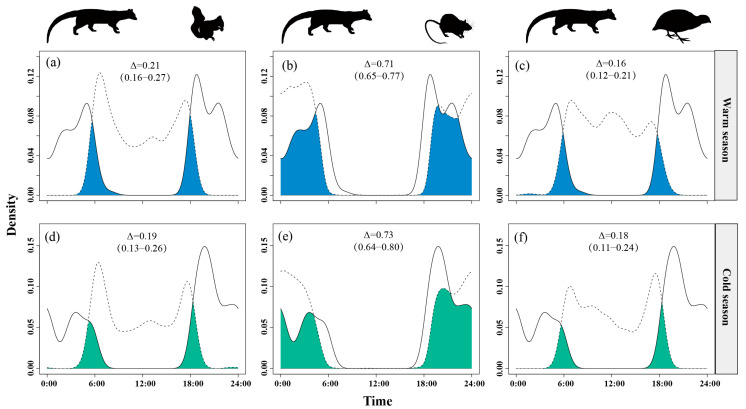
The temporal overlap between small Indian civets (solid curves) and their potential prey (dashed curves) in the warm (**a**–**c**) and cold (**d**–**f**) seasons in Fujian Liangye Mountain National Nature Reserve. Shaded area denotes the overlapping time under the two density curves (Δ).

Temporal overlap between small Indian civets and nocturnal rats remained consistently high across seasons, ranging from Δ = 0.68 in the warm season to Δ = 0.73 in the cold season. Overlap with Pallas’s squirrels was moderate and stable (Δ = 0.18–0.23), while overlap with Chinese bamboo partridges was consistently low (Δ = 0.16–0.20) in both seasons (Figure 8).

### 3.4. Spatial Overlap

**Figure 9:**
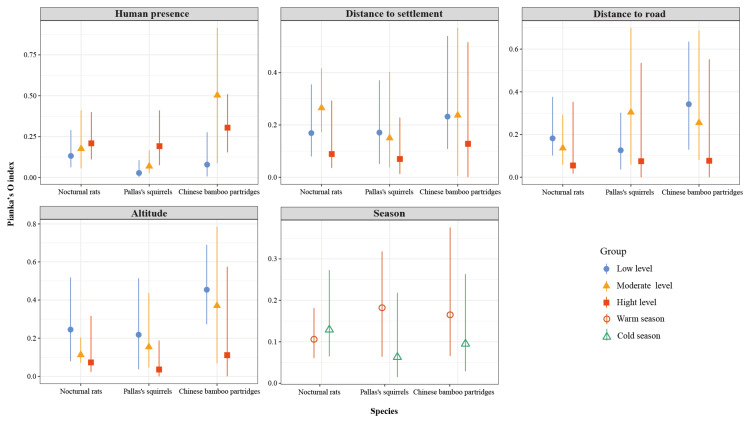
Spatial overlap (Pianka’s index and 95% confidence intervals) between small Indian civets and their potential prey under different human disturbances and different altitudes and seasons in Fujian Liangye Mountain National Nature Reserve. Pianka’s index ranges from 0 (no overlap) to 1 (complete overlap).

Spatial overlap between small Indian civets and their potential prey varied with different types and intensities of human disturbance. Specifically, spatial overlap between small Indian civets and both nocturnal rats and Pallas’s squirrels increased with rising human presence, whereas overlap with Chinese bamboo partridges peaked at moderate levels of human presence (Figure 9). Conversely, spatial overlap between small Indian civets and Chinese bamboo partridges peaked at moderate human presence. Regarding proximity to settlements, spatial overlap between small Indian civets and both nocturnal rats and Chinese bamboo partridges was highest at moderate distances, whereas overlap with Pallas’s squirrels decreased as the distance from settlements increased. Overlap between small Indian civets and nocturnal rats was particularly pronounced at moderate settlement distances. Along road distance gradients, spatial overlap between small Indian civets and nocturnal rats and Chinese bamboo partridges decreased with increasing distance from roads, while overlap with Pallas’s squirrels peaked at moderate road distances. Additionally, spatial overlap between small Indian civets and all three prey species decreased with increasing altitude. Seasonally, spatial overlap between small Indian civets and Pallas’s squirrels and Chinese bamboo partridges was significantly higher in the warm season compared to the cold season, whereas overlap with nocturnal rats was slightly higher during the cold season (Figure 9).

### 3.5. Spatiotemporal Overlap

The spatiotemporal overlap between small Indian civets and nocturnal rats was higher than that of the other two potential prey species under different human disturbances, altitudes and seasons (Table 2). Specifically, overlap between the small Indian civets and both nocturnal rats and Pallas’s squirrels increased with higher human presence, whereas overlap with Chinese bamboo partridges peaked at moderate human presence. Regarding settlement proximity, spatiotemporal overlap between the small Indian civets and both Pallas’s squirrels and Chinese bamboo partridges decreased as distance from settlement increased, whereas overlap with nocturnal rats was highest at moderate distances. Along gradients of road proximity, overlap between the small Indian civets and both nocturnal rats and Chinese bamboo partridges decreased with increasing distance from roads, while overlap with Pallas’s squirrels was highest at moderate distances. With increasing altitude, spatiotemporal overlap between the small Indian civets and nocturnal rats and Chinese bamboo partridges declined; conversely, overlap with Pallas’s squirrels peaked at higher altitudes. Additionally, overlap between the small Indian civets and both Pallas’s squirrels and Chinese bamboo partridges was significantly higher in the warm season compared to the cold season, whereas overlap with nocturnal rats was greater in the cold season (Table 2).

## 4. Discussion

Our study provides the first comprehensive investigation of predator–prey interactions between small Indian civets and their prey in the southern Wuyi Mountains. We demonstrated that both predators and prey adjust their spatiotemporal patterns in response to human disturbances, supporting the concept of anthropogenic landscapes as partial shields for prey species. However, these interactions were scale-dependent, with temporal refuges not necessarily translating to reduced spatial overlap.

We found that, except for nocturnal rats, the other three species exhibited a pronounced crepuscular activity pattern. The crepuscular activity pattern of small Indian civets in our study area differs from the more nocturnal patterns reported elsewhere [28,32,36], highlighting the behavioral plasticity of this species. This plasticity likely enabled the context-dependent shifts we observed in response to gradients of human disturbance, altitude, and season [34].

However, we also found that in areas with high human presence, the small Indian civets advanced their morning activity peak, whereas in areas with moderate human presence, the morning peak was delayed. But at locations close to settlement and at low altitudes, small Indian civets tended to delay the onset of the morning peak while advancing the timing of the evening peak. Areas at lower altitudes and closer to settlements were generally more accessible to humans and experienced higher levels of human activity. Wang et al. [27] and Zhang et al. [30] also found that small Indian civets significantly avoided areas with high human activity by adjusting their activity timing. We found that Pallas’s squirrels and Chinese bamboo partridges increased their daytime activity intensity in areas closer to roads and settlements, supporting our hypothesis that roads and settlements would provide refuges for prey [19,21]. However, in different habitats, nocturnal rats showed little variation in their activity rhythms, indirectly indicating that they possess strong adaptability and that their activity rhythms were relatively conservative. The Pallas’s squirrels and the Chinese bamboo partridge reduced encounters with both the small Indian civets and human activities by shifting their activity peaks to different times [52].

The temporal overlap between small Indian civets and their potential diurnal prey was lowest in areas that were close to roads, had high human presence, and were located at low altitude, suggesting that diurnal prey are released from predation pressure in these locations [21]. In the reserve, human activities such as farming and settlements are primarily concentrated in low-altitude areas. Thus, our findings provide partial support for the human shield hypothesis. Gaynor et al. [53] also stated that the emergence of human shields was highly context-dependent and influenced by ecological factors and human disturbance. In our study areas, the extinction of large carnivores has positioned the small Indian civets and leopard cats (*Prionailurus bengalensis*) as the apex predators within the local food web [40]. The leopard cats are slightly heavier than the small Indian civets, which might explain why the small Indian civets tend to be more active close to settlements—partly to avoid competition with leopard cats and partly to access food from human-generated waste, leading to reduced temporal overlap with diurnal prey [19].

We found that the temporal overlap between the small Indian civets and their potential prey varied with changes in human activity; for example, the highest overlap with all three potential prey species occurred under moderate levels of human presence and at greater distances from settlements. This highlights the complexity of human disturbance effects on predator–prey relationships and suggests that disentangling the impacts of different types of human activity is essential for informing effective conservation and management strategies [8,11]. Although the temporal overlap between the small Indian civets and their three potential prey species varied with human activity, altitudes and seasons, it was consistently highest with nocturnal rats. This suggests that the activity patterns and dietary preferences of small Indian civets may be relatively conservative [34], reflecting a trade-off strategy between maximizing access to prey and minimizing potential threats from daytime human disturbance [36,43].

Spatial overlap is another essential prerequisite for interspecific predation [12]. Human activities can modify the degree of spatial overlap between predators and their prey [7]. In this study, we found that in areas with higher levels of human disturbance—such as higher human presence, closer proximity to settlements and roads, and lower altitude—the small Indian civets exhibited increased spatial overlap with the three potential prey species, indicating an intensification of interspecific interaction strength and far-reaching influences on the community. At this spatial scale, we did not find evidence that settlements and roads provide refuges for diurnal prey and thereby reduce potential predation pressure on them, which does not support our hypothesis. One possible reason is that, with increasing human disturbance, the availability of other food resources for the small Indian civets, such as amphibians and birds, may decline [54,55], thereby intensifying its spatial overlap with the three potential prey species examined in this study. On the other hand, these prey species may be more inclined to inhabit areas with higher human activity to access food resources, such as household waste [19]. In turn, the small Indian civets may also increase their spatial overlap with these prey species to enhance predation opportunities. Ji et al. [43] also found that, with increasing human disturbance intensity, leopard cats increased their predation on diurnal squirrels and nocturnal rats. Future studies could use fecal DNA analysis to compare whether the dietary composition of the small Indian civets differs under varying levels of human disturbance.

We found that in the warm season, the small Indian civets increased their spatial overlap with diurnal prey, whereas in the cold season, they increased their overlap with nocturnal rats. Human activities such as tourism and non-timber forest product harvesting (e.g., mushroom picking) generally avoid the extreme heat of the warm season, occurring more frequently in the cooler cold season [40]. Due to the lower level of human activity in the warm season, avoidance of humans by the small Indian civets was correspondingly reduced, which may have led to increased predation on diurnal prey during dawn and dusk. In addition, most plants produce mature seeds and fruits in the warm season [39], providing abundant food resources for diurnal prey. Food obtained from Pallas’s squirrels and Chinese bamboo partridges was more efficient and energy-saving to acquire, owing to their wider distribution and greater energetic return, as they weigh roughly twice as much as nocturnal rats according to our live-trap survey (Ji, unpublished data). In this study, Pianka’s index was used to quantify spatial overlap. In future analyses, it will be necessary to incorporate habitat covariates and occupancy models to identify the main drivers influencing the distributions and interactions of the small Indian civets and their potential prey.

We used the product of temporal overlap and spatial overlap to represent the probability of the small Indian civets selecting potential prey in space and time [51]. The spatiotemporal overlap results consistently indicated a stronger preference for nocturnal rats, but prey selection among the three potential prey species varied under different levels of human disturbances, altitudes and seasons. However, the types and intensities of human disturbance on predator–prey interaction have often been overlooked in previous studies [4]. We found that in areas with high human disturbance—such as higher human presence, closer to settlements and roads, and low altitudes—the small Indian civets increased their spatiotemporal overlap with all three potential prey species. This suggests that human activities may potentially intensify interspecific predation, which could impact community structure and ecosystem stability through cascading effects [6,8,56]. The same results were also found in Manas National Park, India; tigers (*Panthera tigris*) and their ungulate prey restricted their activities to avoid humans, which in turn increased their spatiotemporal overlap with each other [57]. In addition, some studies suggest that human disturbance does not affect predator–prey spatiotemporal overlap, as their net interactions tend to be relatively conservative [15]. The differences in research findings may be related to factors such as wildlife tolerance to human disturbance in different ecosystems and whether human disturbance is perceived as a threat [8]. This also underscores the importance of considering both the type and intensity of human disturbance in future studies, as low-intensity disturbance or certain disturbance types may not necessarily affect predator–prey interspecific relationships.

However, an important methodological limitation must be considered when interpreting our findings. Our study relied exclusively on camera traps deployed at ground level. While this method effectively captures the terrestrial activity of small Indian civets and nocturnal rats, it is likely to underrepresent the arboreal and aerial activity of Pallas’s squirrels and Chinese bamboo partridges. Consequently, our data primarily reflect predator–prey interactions at the terrestrial level. The temporal activity patterns we documented for the diurnal prey are those associated with their ground-based foraging or movement. It remains possible that a portion of their activity, particularly resting, vigilance, or foraging in the trees, was not fully captured. This sampling bias means that the true extent of spatiotemporal overlap and avoidance between the small Indian civets and their arboreal prey may not be completely quantified. Our results are therefore a conservative estimate of these interactions, particularly in the vertical dimension. Future studies employing a combination of ground- and arboreal-mounted camera traps would be essential to provide a more comprehensive, three-dimensional understanding of these predator–prey dynamics and to validate the patterns observed here.

## 5. Conclusions

A substantial body of research has shown that human activities can alter wildlife behavior by shifting activity patterns, modifying habitat selection, and influencing food resource use, which in turn affects predator–prey relationships within communities [5,13,16,18]. However, species differ in their tolerance and responses to the type and intensity of human activities [4,22], potentially leading to uncertainty in how predator–prey interactions change. Quantifying these interspecific relationships is therefore of great importance for informing species conservation and protected area management. We found that all species adjusted their activity timing under different types and intensities of disturbance, either advancing or delaying their activity peaks, to achieve temporal segregation. At the temporal overlap scale, the small Indian civets and their potential prey were consistent with our hypothesis that higher levels of human disturbance allowed diurnal prey to be released from predation pressure. However, at both spatial and spatiotemporal scales, higher human disturbance increased the spatial and spatiotemporal overlap between the small Indian civets and the three potential prey species. Our results also revealed a consistent effect of the disturbance types examined in this study on the predator–prey relationships between the small Indian civets and their potential prey. One potential explanation is that in systems with dominant competitors like leopard cats and high human disturbance, subordinate predators such as small Indian civets may be forced into suboptimal habitats near human activity to avoid competition. This study provides a preliminary investigation into the spatiotemporal relationships between the small Indian civet and three potential prey species. Future research should incorporate methods such as fecal DNA analysis and occupancy modeling to further elucidate the habitat and food resource selection of small Indian civets and their response to human activities.

## Figures and Tables

**Table 1:** Monitoring data of small Indian civets and their potential prey in Fujian Liangye Mountain National Nature Reserve.

Species	Activity Pattern	Weight	Number of Independent Photos	Camera Sites with Detection	RAI
Small Indian civets	nocturnal	1600–4000 g	307	46	0.50
Nocturnal rats	nocturnal	20–2000 g	10,429	179	17.12
Pallas’s squirrels	diurnal	280–420 g	629	93	1.03
Chinese bamboo partridges	diurnal	200–342 g	518	54	0.85

**Table 2:** Spatiotemporal overlap between small Indian civets and their potential prey under different human disturbances, altitudes and seasons in Fujian Liangye Mountain National Nature Reserve.

Pairwise Species	Spatiotemporal Overlap		
	Low	Moderate	High
Human presence			
SIC-NR	0.083	0.130	0.150
SIC-PS	0.006	0.020	0.040
SIC-CBP	0.017	0.116	0.040
Distance to settlement			
SIC-NR	0.115	0.207	0.069
SIC-PS	0.034	0.026	0.020
SIC-CBP	0.037	0.031	0.028
Distance to road			
SIC-NR	0.126	0.095	0.045
SIC-PS	0.023	0.088	0.014
SIC-CBP	0.051	0.048	0.012
Altitude			
SIC-NR	0.167	0.082	0.055
SIC-PS	0.050	0.031	0.107
SIC-CBP	0.082	0.074	0.023
Season	Warm season	Cold season	
SIC-NR	0.075	0.094	
SIC-PS	0.038	0.012	
SIC-CBP	0.026	0.017	

Note: SIC—small Indian civets; NR—nocturnal rats; PS—Pallas’s squirrels; CBP—Chinese bamboo partridges.

## Data Availability

The data are available upon reasonable request from the corresponding author (Chengpeng Ji).

## Supplementary Materials

The following supporting information can be downloaded at https://www.mdpi.com/article/10.3390/ani15213121/s1, Figure S1: Differences in the daily activity of small Indian civets and their potential prey under different levels of human presence in Fujian Liangye Mountain National Nature Reserve; Figure S2: Differences in the daily activity of small Indian civets and their potential prey under different distance to settlement in Fujian Liangye Mountain National Nature Reserve; Figure S3: Differences in the daily activity of small Indian civets and their potential prey under different distance to road in Fujian Liangye Mountain National Nature Reserve; Figure S4: Differences in the daily activity of small Indian civets and their potential prey under different altitude in Fujian Liangye Mountain National Nature Reserve; Figure S5: Differences in the daily activity of small Indian civets and their potential prey under different season in Fujian Liangye Mountain National Nature Reserve.
