# Supplementary material for: The Human Shield in Time but Not in Space: Scale-Dependent Responses of Small Indian Civet–Prey Interactions to Anthropogenic Disturbance
Source: Animals (Basel). 2025 Oct 28;15(21):3121. doi: 10.3390/ani15213121 (PMC12608993; doi:10.3390/ani15213121)
Supplement: Supplementary file 1 [file animals-15-03121-s001.zip › animals-3917448-supplementary.pdf]

The Human Shield in Time but Not in Space: Scale-Dependent Responses of Small Indian Civet–Prey Interactions to Anthropogenic Disturbance

Supporting information

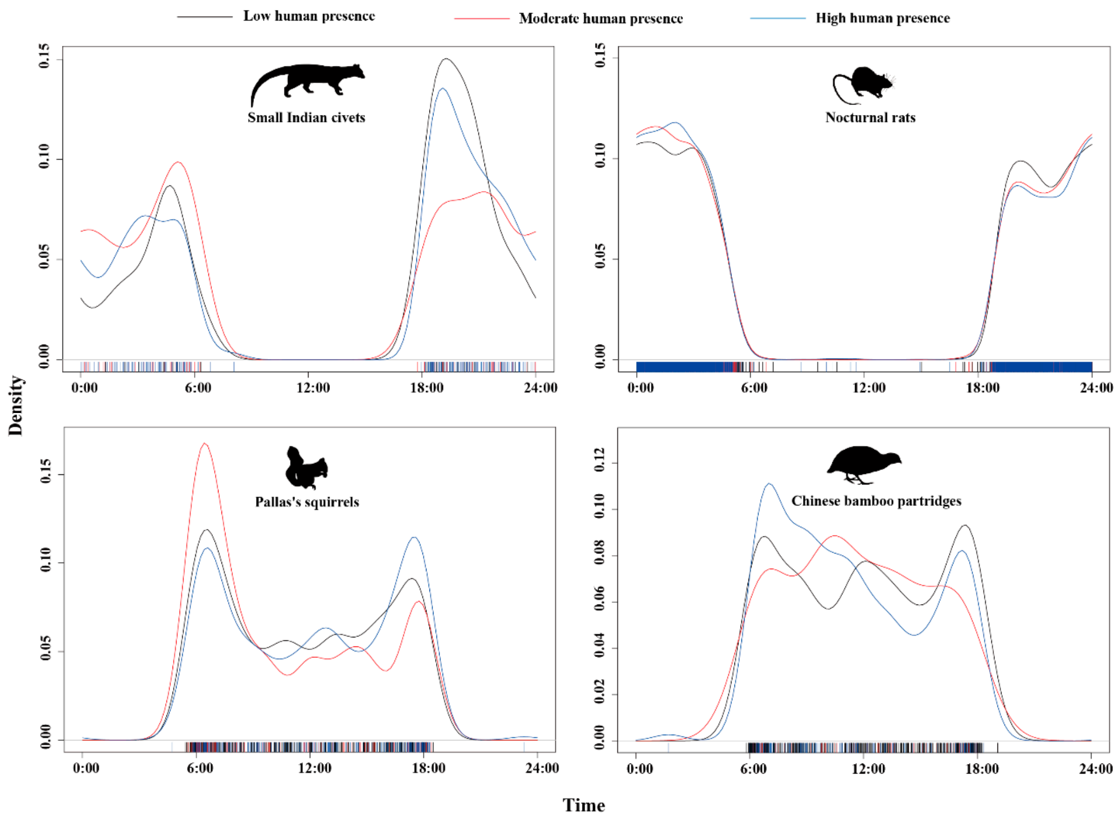

Figure S1. Differences in the daily activity of small Indian civets and their potential prey under different levels of human presence in Liangyeshan National Nature Reserve.

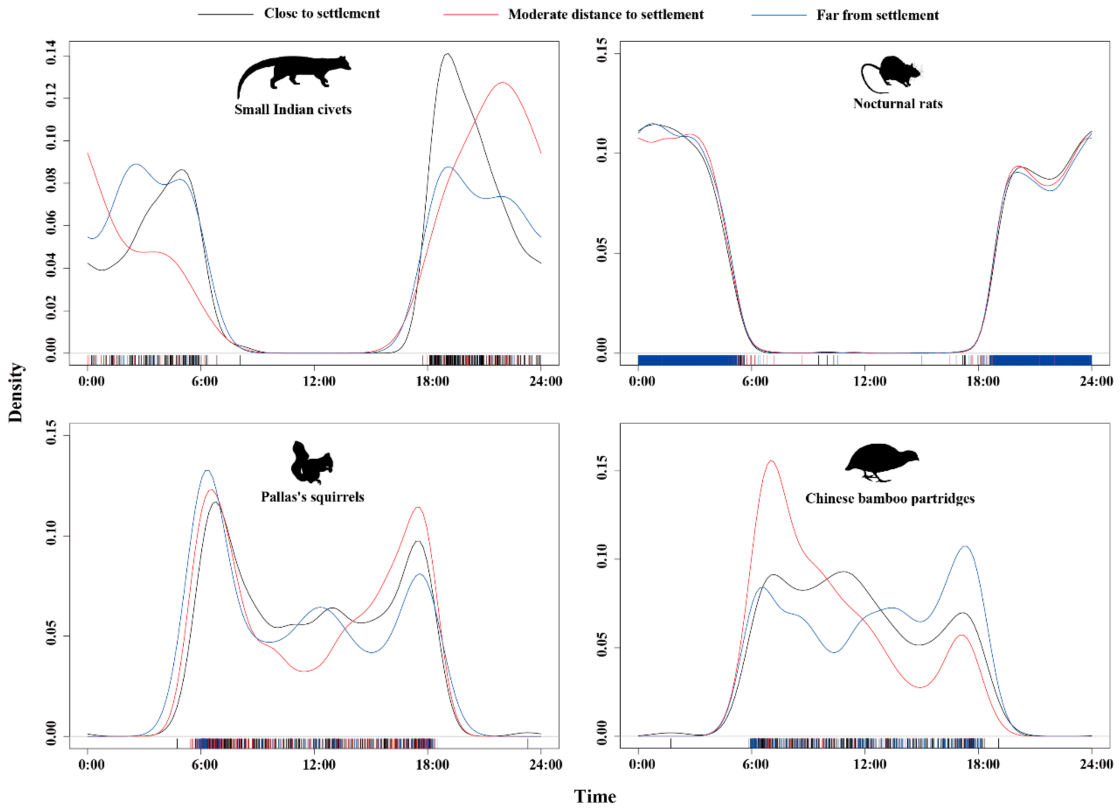

Figure S2. Differences in the daily activity of small Indian civets and their potential prey under different distance to settlement in Liangyeshan National Nature Reserve.

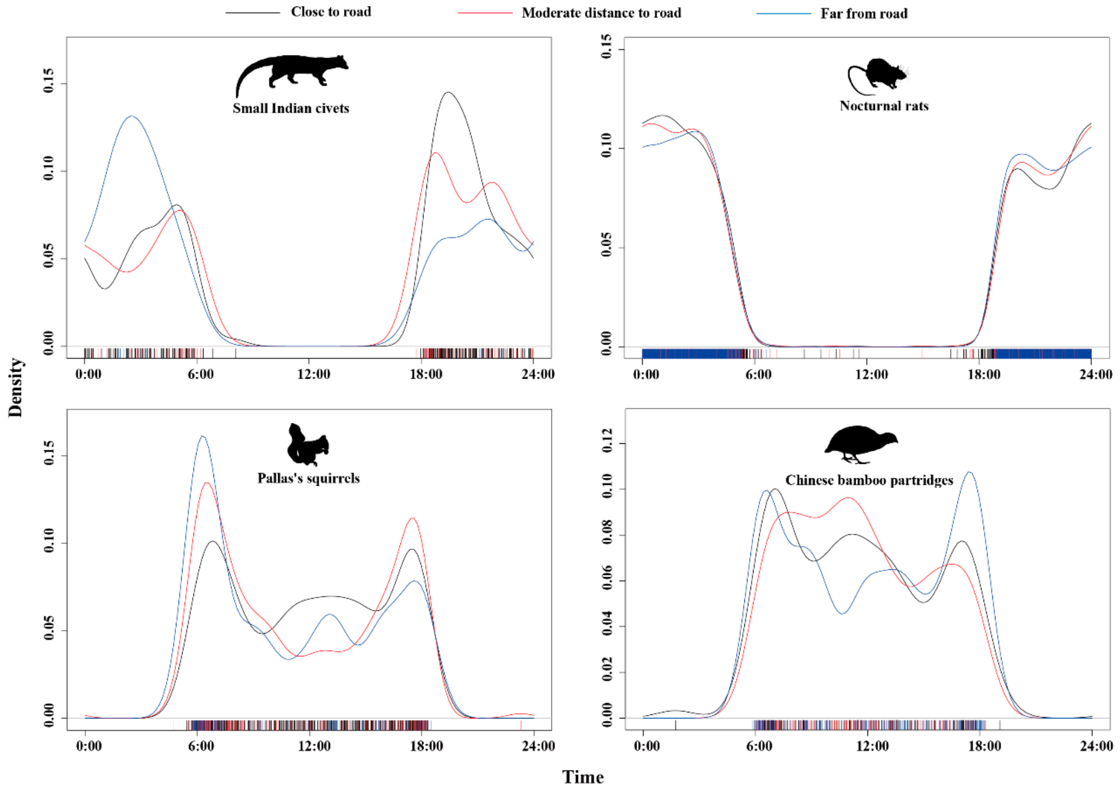

Figure S3. Differences in the daily activity of small Indian civets and their potential prey under different distance to road in Liangyeshan National Nature Reserve.

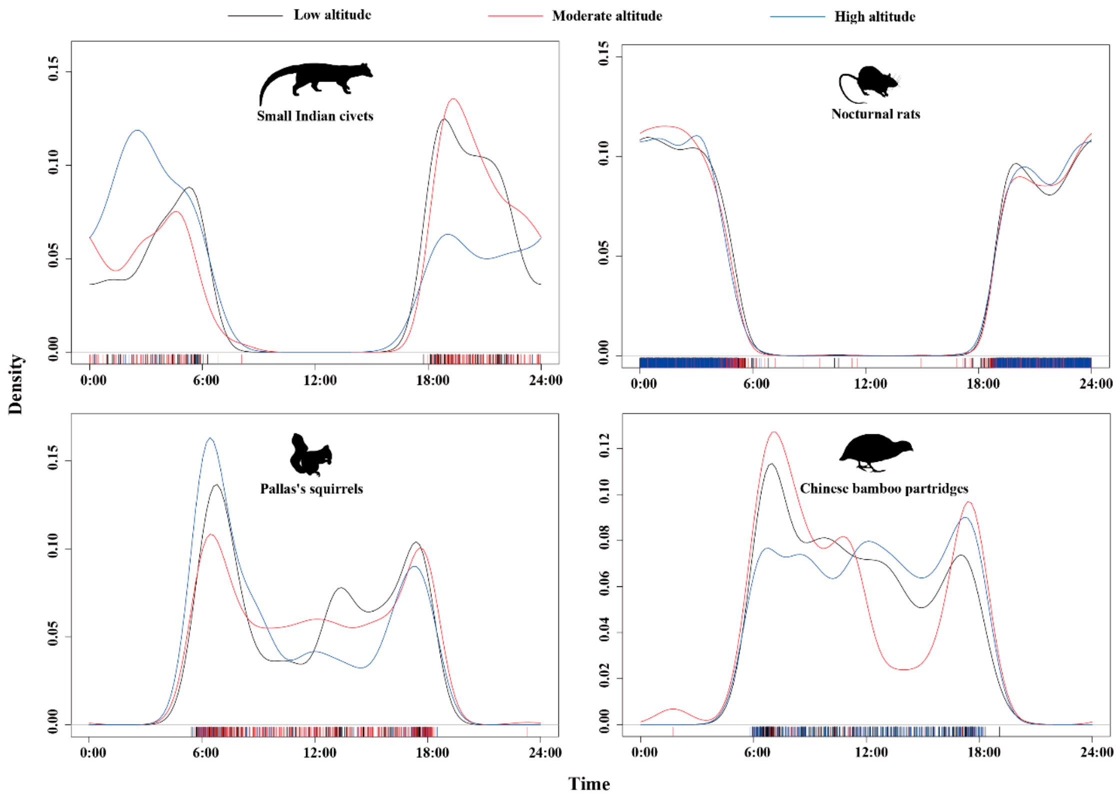

Figure S4. Differences in the daily activity of small Indian civets and their potential prey under different altitude in Liangyeshan National Nature Reserve.

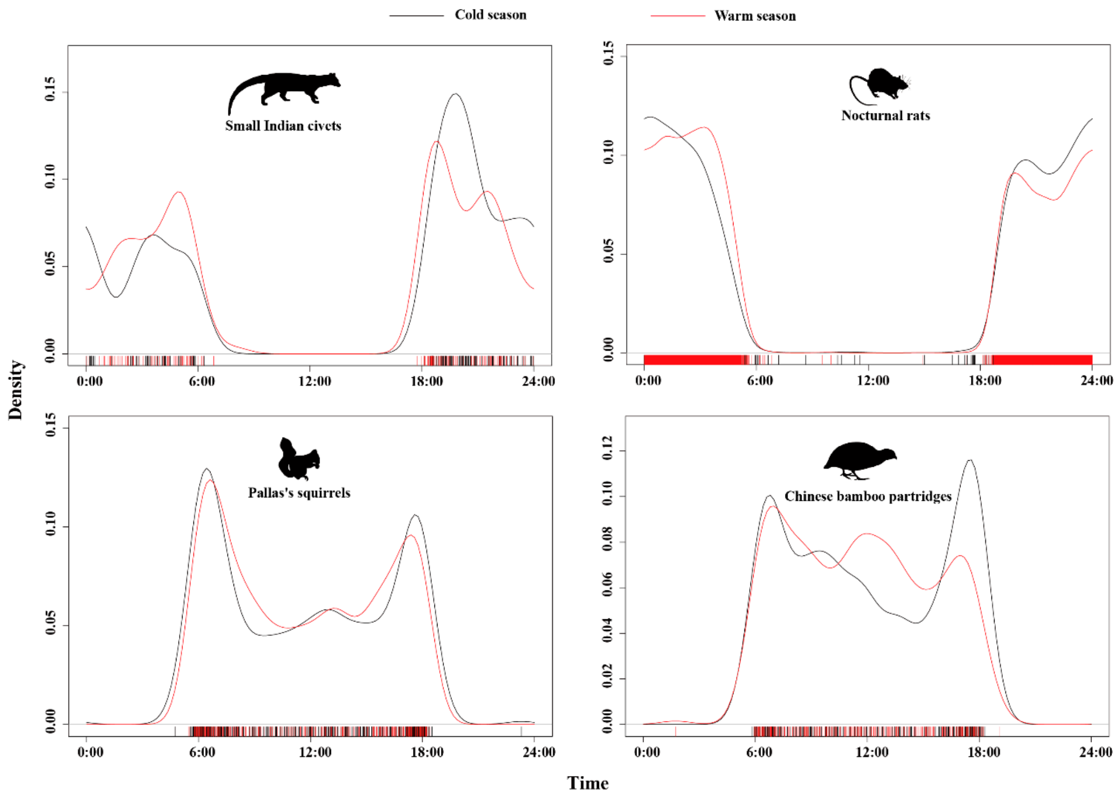

Figure S5. Differences in the daily activity of small Indian civets and their potential prey under different season in Liangyeshan National Nature Reserve.
